# Antimicrobial Activity of Leaf Aqueous Extract of *Schinus polygamus* (Cav.) Cabrera against Pathogenic Bacteria and Spoilage Yeasts

**DOI:** 10.3390/plants13162248

**Published:** 2024-08-13

**Authors:** Andrea Acuña-Fontecilla, Julio Bruna, María Angélica Ganga, Liliana Godoy

**Affiliations:** 1 Departamento en Ciencia y Tecnología de los Alimentos, Facultad Tecnológica, Universidad de Santiago de Chile, Alameda 3363, Estación Central, Santiago 9170002, Chile; 2 Departamento de Fruticultura y Enología, Facultad de Agronomía y Sistemas Naturales, Pontificia Universidad Católica de Chile, Santiago 8940000, Chile

**Keywords:** *Schinus polygamus*, antimicrobial, food contamination

## Abstract

The antimicrobial activity of an aqueous extract of the leaves of *Schinus polygamus* (cav.) Cabrera against microorganisms of food importance was evaluated. First, the leaf aqueous extract of *Schinus polygamus* was characterized, quantifying hydroxycinnamic acids and phenolic compounds. Then, a battery of strains was tested, including *Escherichia coli* ATCC 25922, *Salmonella Typhimurium* ATCC 14028, and *Listeria monocytogenes* ATCC 13932. Also, we tested wine spoilage yeasts such as *Brettanomyces bruxellensis* LAMAP2480, *B. bruxellensis* LAMAP1359, *B. bruxellensis* CECT1451, and *Pichia guilliermondii* NPCC1051. Tests were conducted using the kinetic curve of growth and cell viability counts. The results indicate that with 10% *v*/*v* of concentrated extract, it is possible to observe growth inhibition of all microorganisms studied, with statistically significant differences during the whole measurement time (70 h for bacteria and 145 h for yeast).

## 1. Introduction

Most foodborne diseases (FBDs) are of microbial origin, making them one of the world’s most important public health problems. The ingestion of contaminated food can lead to multi-organ failure, including cancer, and therefore represents a considerable burden of disability and mortality [1], which is why microbiological control is one of the important factors to be safeguarded in the food industry. This control is mainly exerted by incorporating chemical antimicrobial compounds into the product. However, despite their usefulness, they do not meet consumer demand, where new trends in food conservation lead to a reduction in preservative levels and/or the use of “naturally occurring” antimicrobial compounds [2] to obtain the freshest produce, close to its original form [3].

Moreover, there is a need to address the problem of resistance to antibiotics and pesticides, which has led to the search for new sources of natural and safe antimicrobial agents that can be classified by their origin [4,5], which can be derived from vegetable, animal, and microbial sources [3].

In most plant species, whether plants, herbs, or spices, there is an enormous biochemical diversity of compounds [6]. This diversity is associated in part with an evolutionary process to acquire mechanisms of defense or adaptation against the attacks of invading microorganisms, insects, and herbivores [7]. These natural compounds of low molecular weight can be obtained from leaves, flowers, bulbs, rhizomes, bark, and fruits, of which an important percentage possesses insecticidal activity, as well as antimicrobial, inhibiting the activity of bacteria, yeasts, and molds [8]. These may be identified as secondary metabolites or hydrolytic enzymes (chitinase or β-1,3-glucanases) and other proteins and are usually grouped according to the chemical substances that constitute them. Their structure includes nitrogenous toxins (alkaloids, cyanogenic glycosides, glucosinolates, toxic amino acids, lectins, and protease inhibitors); terpenes (sesquiterpene lactones, cardiac glycosides, saponins); and polyacetylenic hydrocarbons, oxalates, and phenolic compounds (tannins, phytoestrogens, flavonoids, and coumarins) [9,10]. Regarding the bioactive action of the aforementioned phenolic compounds, it has been described that they can permeabilize and destabilize the plasma membrane, in addition to inhibiting extracellular enzymes [11]. In particular, the antimicrobial effect of phenolic compounds is related to their chemical characteristics of weak organic acids. In this way, the undissociated forms of these acids are capable of crossing the cell membrane by passive diffusion, lowering the intracellular pH by breaking the cell membrane, causing the release of essential intracellular constituents and causing cell death [12,13,14,15].

Due to the high energy demand involving synthesis, plant metabolism is channeled to one type of secondary compound, depending on the available resources. That is, the groups have a restricted distribution in the plant kingdom, are synthesized in small amounts, and are not widespread, often being restricted to a certain plant genus, family, or even some species [7]. A wide variety of species belonging to the family Anacardiaceae have been investigated for their potential against certain diseases [9,16], for exhibiting insecticidal properties [17,18], and as antimicrobials against fungi and bacteria [2,5,19]. It has been described that the leaf oil of *Schinus polygamus* species has an important inhibitory effect against *Escherichia coli* ATCC25922 and *Pseudomonas aeruginosa* ATCC10145 [20,21]. In Chile, it is used by folk medicine for the cleaning of wounds [22] due to the antiseptic properties of the infusion of its leaves. Also, as a traditional artisan practical, small Atacama winemakers use it for cleaning and sanitizing wine storage barrels. In this way, the antimicrobial effect of aqueous extracts of leaves of *S. polygamus* (Cav.) Cabrera (Molle), against pathogenic food bacteria and spoilage yeasts, was evaluated in the present study.

## 2. Results and Discussion

### 2.1. Identification of Schinus polygamus (Cav.) Cabrera

This species belongs to the Anacardiaceae family, it is native to South America, and its presence has been reported in South America (Argentina, Brazil, Peru, Uruguay, and Chile) and other continents [20,23]. In Chile, it occurs between the Atacama and Los Ríos regions [24]. Locally in the Atacama region, it is known as “Molle”. For our study, it was visually identified according to its exact geographic location based on the description of the characteristics of its branches, leaves, bark, fruits, etc.

According to popular medicine, the infusion of leaves of this plant has been used to clean wounds due to its anti-inflammatory, analgesic, and antiseptic properties [22]. In addition, Gonzalez et al. [25] described the high antimicrobial activity of the essential oil from the leaves of this species against *Bacillus cereus*. Other studies carried out with this plant’s essential oils and dichloromethane-based extract have shown that it would also have antimicrobial activity against *Staphylococcus aureus*, *Micrococcus flavus*, *Bacillus subtilis*, *P. aeruginosa*, *Candida albicans*, and *Aspergillus niger* [20,22]. In addition, Guetchueng et al. [19] showed that both the leaves and the branches would have antimicrobial activity against *E. coli*, *S. aureus*, *P. aeruginosa*, and *C. albicans*, among others.

### 2.2. Quantification of Classic Antimicrobial Compounds in Natural Plants

**Figure 1:**
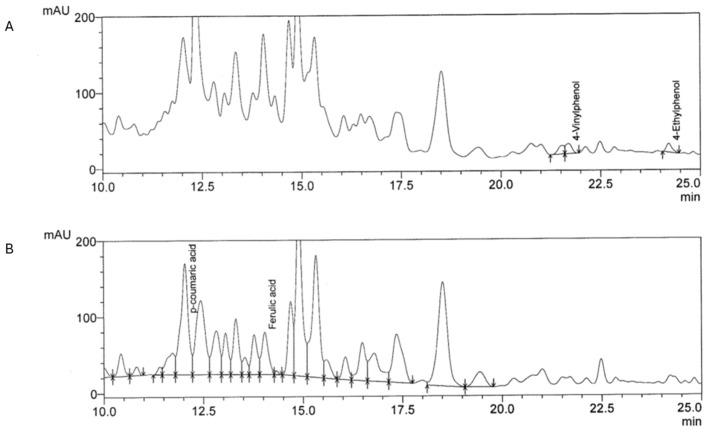
HPLC chromatograms of volatile phenols (**A**) and hydroxycinnamic acids (**B**) present in *S. polygamus* aqueous extract.

The chromatograms of volatile phenols (A) and hydroxycinnamic acids (B) contained in *S. polygamus* aqueous extract, measured by HPLC at 280 nm and 320 nm, respectively, are shown in Figure 1.

We detected ferulic acid, *p*-coumaric acid, 4-vinylphenol, and 4-ethylguaiacol, but not caffeic acid or 4-ethylguaiacol. Table 1 shows the results of the quantification of hydroxycinnamic acids in the aqueous extract of *S. polygamus*.

Volpini-Klein et al. [26] detected the presence of caffeic acid and ferulic acid in a hydroalcoholic extract from the leaves of *S. molle*. In addition, El-Massry et al. [27] found caffeic acid and coumaric acid in an ethanolic extract from fresh leaves of *Schinus terebinthifolius*. Our results show that the aqueous extract of *S. polygamus* contains ferulic acid and *p*-coumaric acid but not caffeic acid. Several authors have shown that there is an influence between the solvents used for the extraction of phenolic compounds as hydroxycinnamic acids, and the type of compound extracted. In this sense, Turkmen et al. [28] demonstrated that by using 50% ethanol, the efficiency of polyphenol extraction increases 2.5 times compared to extraction with pure water. Similarly, Tan et al. [28] confirmed that the mixture of water and ethanol is better for the extraction of phenols than distilled water, although they used a mixture of ethanol and water at 60%. This demonstrates that the amount and type of phenolic compounds extracted from the plant are influenced by the solvent used to prepare the extract, the parameters of the extraction process, in addition to the anatomical parts of the plants used [29].

Other compounds were also identified in the aqueous extract (Table 2).

Tlili et al. [30] extracted phenolic compounds from *S. molle* with methanol from the fruit, and found that the main components were gallic acid, vanillic acid, coumarin and luteolin. da Silva et al. [31] extracted leaves of *S. terebinthifolius* Raddi with methanol and identified the presence of quercetin and luteolin. Of these identified compounds, we detected, among others, naringenin 4′-O-glucoside, luteolin 5,7,3′, 4′ tetramethyl ether, and quercetin 3′ glucoside, possibly more soluble in water than luteolin and quercetin. Also, both in our work and in that of Tlilii et al. [30], rutin was identified as a flavonoid glucoside, which is soluble in water and methanol.

Although there is a difference in the method of extraction, the variation in the composition of phenolic compounds may be due to the composition of each part of the plant, since although they are from the same species, their geographical location may present differences in their chemical composition [32]. Similar observations were made by Garzoli et al. [33], who indicated that one of the most important factors that influences the variation in the chemical composition from plant to plant of the same species is its genotype, as well as the climatic conditions and availability of water and soil nutrients.

**Figure 2:**
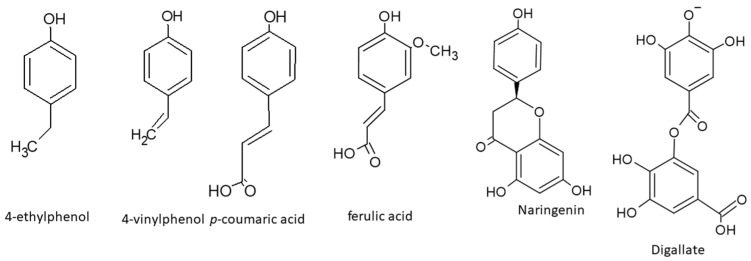
Structures of main identified compounds in *S. polygamus* leaf aqueous extract.

The main compounds identified in *S. polygamus* leaf aqueous extract are illustrated in Figure 2.

### 2.3. Effect on the Growth of Microorganisms

Nunes et al. [34] demonstrated that different types of extraction carried out on plant leaves would allow for obtaining different antimicrobial compounds. This is how they observed that the aqueous extraction of *Erica australis* L. leaves had an antimicrobial effect on a greater number of tested bacterial species such as *E. faecalis*, *B. cereus*, *E. coli*, *S. aureus*, and *L. monocytogenes* compared to an organic extract. This suggests that compounds with greater antimicrobial activity would have a greater affinity for water, which is why they would be much more polar than those extracted with organic solvents.

Various studies have reported that a wide range of microorganisms are sensitive to hydroxycinnamic acids, but the effects depend on the concentration to which they are exposed [10,35,36,37]. In the same way, it has been described that ferulic acid and *p*-coumaric acid would be the most inhibitory [14,38,39,40]. Likewise, isoflavones such as genistin and genistein (and respective isomers) have been reported to have antibacterial activity [41]. The effect would be given by an alteration that increases the fluidity of the plasmatic membrane [42,43]. Also, both naringenin and quercetin have been shown to significantly inhibit bacterial motility (associated with the virulence capacity of bacteria) [42]. Naringenin has been described to have antimicrobial potential against Gram-positive (*S. aureus*, *Listeria innocua*, *B. subtilis*, *Lactococcus lactis*, and *Enterococcus faecalis*) and Gram-negative (*E. coli*, *Salmonella enterica*, and *Pseudomonas putida*) bacteria, as well as the yeast *Saccharomyces cerevisiae* [44].

**Figure 3:**
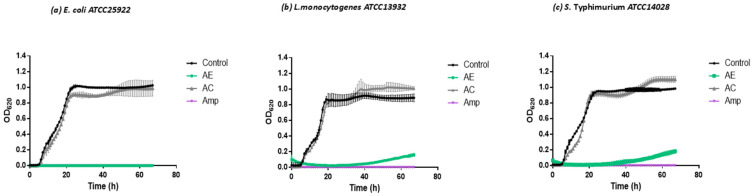
Effect of aqueous extract of *S. polygamus* on the growth of bacteria. (**a**) *E. coli* ATCC25922; (**b**) *S. Typhimurium* ATCC14028; (**c**) *L. monocytogenes* ATCC13932. Control = medium of culture; AE = aqueous extract (10% water plus medium); AC = aqueous control (10% water plus medium); Amp = medium plus ampicillin.

Our study used aqueous extracts of *S. polygamus* (Cav.) Cabrera. The extract was tested on yeast and bacteria species of interest in the food industry. In the case of bacteria, the AE had a negative effect on the growth kinetics of all the strains tested (Figure 3).

Figure 3a shows how the AE inhibited the growth of *E. coli* ATCC25922 during the 64 h of analysis. An equivalent effect was observed in the positive control culture that contained ampicillin as an antibiotic. In the case of *L. monocytogenes* and *S. Typhimurium* (Figure 3b,c), it was shown that at 40 h in both cases, there was an increase in optical density (OD). This increase was significantly lower compared to the cultures where only the microorganisms were grown with a culture medium without any growth inhibitor.

To determine if the water in which the extract of *S. polygamus* (Cav.) Cabrera being diluted would affect the growth of the bacteria tested by diluting the nutrients in the culture medium, a control (AC) was carried out, which corresponded to the addition of sterile distilled water in a volume of 10% of the total volume of the culture (similar to the dilution used in the case of the AE). Figure 3 shows that the addition of this amount of water did not affect the growth of the tested bacteria; therefore, the inhibition exerted by the *S. polygamus* extract was caused by the antimicrobial compounds extracted from the plant.

**Figure 4:**
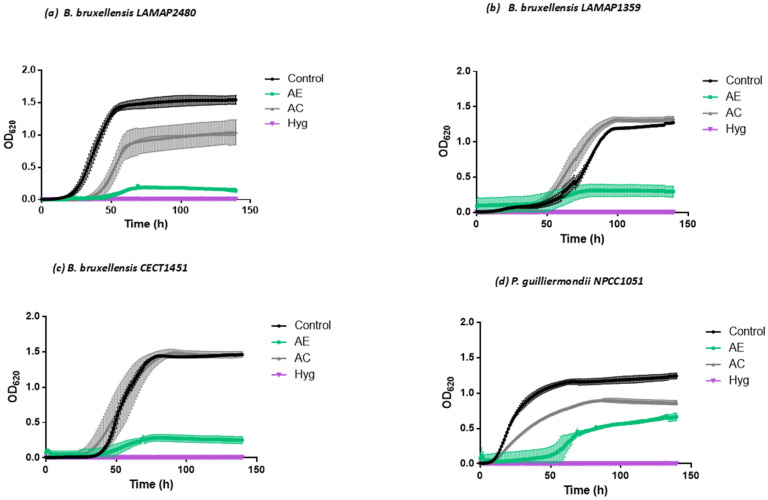
Effect of aqueous extract of *S. polygamus* on the growth of spoilage yeasts. (**a**) *B. bruxellensis* LAMAP2480; (**b**) *B. bruxellensis* LAMAP 1359; (**c**) *B. bruxellensis* CECT1451; (**d**) *P. guillermondii* NPCC1051. Control = medium of culture; AE = aqueous extract; AC = aqueous control (10% water); Hyg = medium plus hygromycin.

As previously mentioned, these aqueous extracts are used by some wine producers in the Atacama region, where the *S. polygamus* leaves were obtained. For this reason, an assay was carried out to determine the effect of the AE on spoilage yeasts of this type of product, such as *B. bruxellensis* and *P. guilliermondii* (Figure 4).

In the presence of the 10% AE in the medium, all tested yeast strains decreased their growth kinetics (Figure 4 and Table S1). This is how it was determined that the specific growth rate was reduced by 88% for the *B. bruxellensis* LAMAP2480 strain, 82% for the *B. bruxellensis* LAMAP1359 strain, and 85% for the *B. bruxellensis* CECT1451 strain compared to the control. In the case of *P. guilliermondii* NPCC1051, the growth rate reduction reached 69%. Additionally, the OD values were statistically lower than the control cultures during the 145 h incubation in all the cases tested.

For its part, the AE caused an increase in the duration of the *lag* phase of the growth curve for the tested yeast strains. For *B. bruxellensis* LAMAP2480, the duration of the *lag* phase was, on average, 1.5 times longer compared to the control condition. In the case of *P. guilliermondii* NPCC1051 it was 5.7 times (Figure 4a,d), this last strain being the most resistant to the antimicrobial effect of the AE (it obtained a higher OD at the end of the experiment) (Table S1). It should be noted that both strains (LAMAP2480 and NPCC1051) showed lower growth than the AC control cultures compared to their respective control cultures. This suggests that the compounds extracted from *S. polygamus* (Cav.) Cabrera would not have the only inhibitory effect on these yeasts.

*B. bruxellensis* LAMAP1359 and CECT1451 (Figure 4b,c) presented a prolonged *lag* phase duration when they were grown in culture medium, and the addition of the AE caused an increase in the value of this parameter by 1.18 times for *B. bruxellensis* LAMAP1359 and 1.33 times for *B. bruxellensis* CECT1451, thus reducing growth kinetics in both strains (Table S1).

Harris et al. [40] observed that in *B. bruxellensis* strains, the specific growth rate was reduced when grown in a culture medium supplemented with 333 mg/L of hydroxycinnamic acids. Likewise, they observed that above 50 mg/L of these acids, the duration of the *lag* phase was prolonged, indicating that the presence of hydroxycinnamic acids in combination decreased yeast growth. However, their growth increased as the concentration of these acids in the culture medium decreased, being able to adapt and metabolize it.

On the other hand, phenols have been described as having antimicrobial activity, so their use could be an efficient control method to increase the shelf life of food [45]. Three mechanisms have been described in which phenolic compounds could affect microbial growth: (a) modification of cell permeability forming cytoplasmic granules and rupture of the cytoplasmic membrane; (b) changes in intracellular functions due to the hydrogen binding of the phenolic compound to enzymes; (c) changes in the morphology of the fungal cell wall (loss of cell rigidity or integrity). There is a subgroup to highlight within the phenolic compounds, such as hydroxycinnamic acids. These are present in the plant cell wall and have been described as natural food preservatives [46] since they can affect the metabolism or inhibit the growth of various organisms, including plants, fungi, and Gram-positive and negative bacteria [47,48]. The effect of these compounds was evaluated by Rodríguez Sauceda [3], who described that the free forms of hydroxycinnamic acids, mainly p-coumaric acid, caffeic acid, and ferulic acid, could inhibit the growth of *E. coli*, *S. aureus*, and *B. cereus*. In addition, it has been reported that these acids can be carbon sources for microorganisms and are degraded through oxidative or non-oxidative metabolic pathways [49]. The non-oxidative metabolic routes of hydroxycinnamic acids imply a decarboxylation of these, becoming hydroxystyrenes. The enzyme that carries out the decarboxylation step is present in many bacteria, fungi, and yeasts. However, the step of reducing hydroxycinnamic acids is restricted to only the species *B. bruxellensis*, *P. guilliermondii*, *Candida versatilis*, *Candida halophila*, and *Candida mannitofaciens* [50,51,52]. Figure 4 shows how these yeasts are affected by their growth curves in the first hours of exposure to the AE, since after approximately 40 h, an adaptation to the growth condition is observed, which does not become significantly equal to the control condition.

Previous work by our research group indicates that one of the identified compounds, *p*-coumaric acid, generates a state of generalized stress, inducing the expression of proton pumps and mechanisms involved in the release of toxic compounds. Furthermore, these mechanisms could be involved in the release of nitrogen compounds, such as amino acids, decreasing the overall concentration and triggering the expression of nitrogen metabolism genes. Likewise, p-coumaric acid also induces oxidative stress, activating the expression of genes such as SOD1, PST2, and PRX1. Finally, this acid triggers a change in cell wall permeability in order to reduce the rate of entry of weak acids into the cell [15].

Other phenolic compounds that have been shown to have antimicrobial activity are tannins and tannic acid. The latter, for example, is inhibitory for *L. monocytogenes*, *E. coli*, *Salmonella Enteritidis*, *S. aureus*, *Aeromonas hydrophila*, and *Streptococcus faecalis* [3]. These phenolic compounds would decrease RNA synthesis and the proteolytic or cellulolytic activity due to their action on microbial enzymes [11]. Also, another explanation could have a relation with the volume used in the culture medium, where the microorganisms could process the aqueous extract during the first 40 h. In addition, according to our results, the constituents of the extract are not lytic to the yeast, affecting only the growth, becoming slower.

The antimicrobial effect, expressed as the MIC of the aqueous plant extract against the test microorganisms, is shown in Table 3.

The data revealed variability in the MIC values. The lowest MIC values (2.5%) were exhibited by water extracts against *B. bruxellensis* LAMAP1359. For bacteria such as *E. coli* and *L. monocytogenes*, the MIC value was 5%. The MIC value for *S. Typhimurium* and yeasts such as *B. bruxellensis* LAMAP2480, *B. bruxellensis* CECT1451, and *P. guilliermondii* NPCC1051 was greater than 5%. Aboalhaija et al. [53] observed that the aqueous extract of *S. polygamus* inhibited the growth of *Citrobacter freundii* and *Enterococcus fecalis* using the lowest concentration (1.56 mg/mL). Also, the effect of the aqueous extract, both on *P. aeruginosa* and *S. aureus*, was similar, as the potential minimum bactericidal concentration (MBC) was measured to be 12.5 mg/mL, and subsequently, the MIC was 6.25 mg/mL. The expected MIC for *E. coli* was 12.5 mg/mL. The least effect of the aqueous extract was noticed on *S. epidermidis*, with an MIC value of 25 mg/mL.

As previously mentioned, naringenin was one of the compounds identified in the AE. The MIC described for naringenin varied between 0.25 mg/mL and 1 mg/mL, suggesting that the action would depend on the target microorganism [44,54]. Also, we detected digallate, which has been reported to inhibit the growth of methicillin-resistant *S. aureus* cultures, with MICs of 0.05 to 0.1 mg/mL and concentrations of 0.8 mg/mL to inhibit the growth of Gram-negative species such as *E. coli*, *Klebsiella pneumoniae*, *Salmonella typhi*, *Proteus mirabilis*, *P. aeruginosa*, and *Serratia marcescens* [55].

Although this result suggests antimicrobial action on the growth of all microorganisms tested, it is not possible to determine that it has a microbicidal and microbiostatic action. Cell viability tests were performed to determine these effects.

### 2.4. Evaluation of the Cell Viability of the Tested Microorganisms

**Figure 5:**
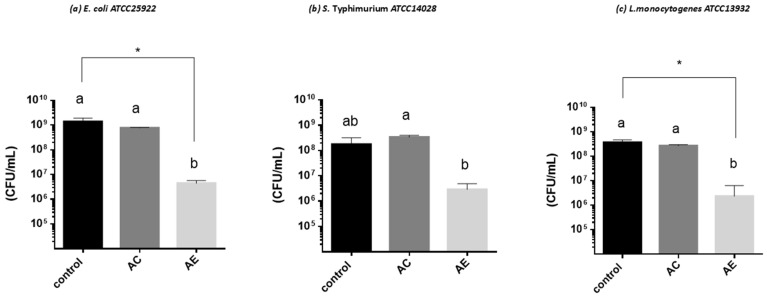
Effect of aqueous extract of *S. polygamus* on cell viability of bacteria. (**a**) *E. coli* ATCC 25922; (**b**) *S. Typhimurium* ATCC14028; (**c**) *L. monocytogenes* ATCC13932. Control = medium of culture; AC = aqueous control (10% water plus medium); AE = aqueous extract (10% extract plus medium). Different letters above each column represent a significant difference (* *p* ≤ 0.05).

**Figure 6:**
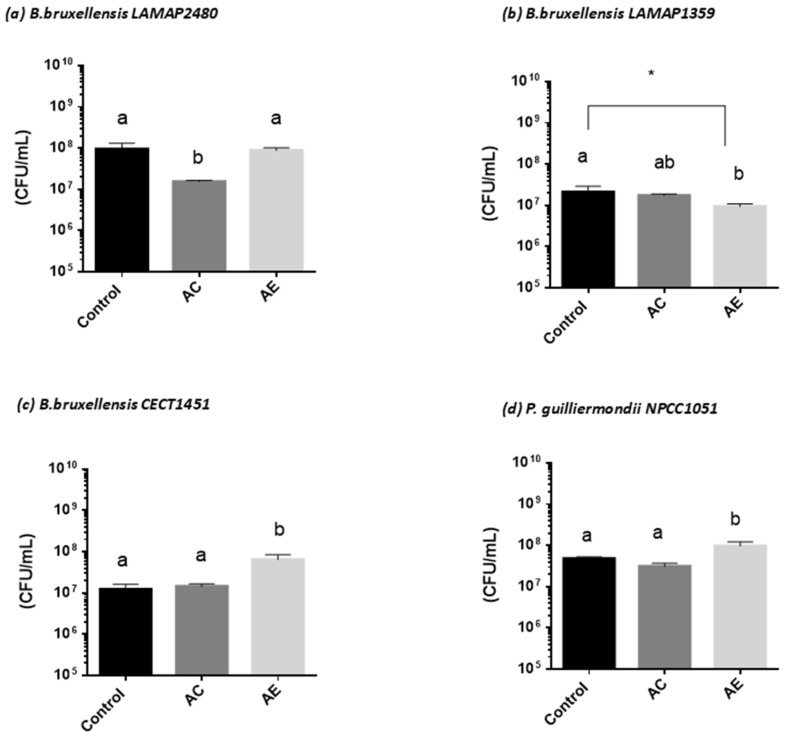
Effect of aqueous extract of *S. polygamus* on cell viability of spoilage yeasts. (**a**) *B. bruxellensis* LAMAP2480; (**b**) *B. bruxellensis* LAMAP 1359; (**c**) *B. bruxellensis* CECT1451; (**d**) *P. guilliermondii* NPCC1051.Control = medium de culture; AC = aqueous control (10% water plus medium); AE = aqueous extract (10% extract plus medium). Different letter above each column represents a significant difference (* *p* ≤ 0.05).

This test allowed for quantifying the in vitro susceptibility of microorganisms to natural substances of plant origin present in the aqueous extract *S. polygamus* (Cav.) Cabrera (Figure 5 and Figure 6).

Most food antimicrobials are chemical compounds that act as bacteriostatics and fungistatics, and they do not manage to destroy microorganisms, so their effectiveness on food is limited [3].

In our work, it was observed that although the AE of *S. polygamus* (Cav.) Cabrera significantly inhibited the growth of the microorganisms studied by reducing growth kinetics, the AE failed to inhibit cell viability completely. Bacteria viability (Figure 5) was affected negatively, observing a decrease of 2 log cycles for *E. coli*, 1 log cycle for *S. Typhimurium*, and 1.5 log cycles for *L. monocytogenes*. During microbial growth, the metabolization of substances in the culture medium is carried out, producing reducing agents that affect microbial growth. However, these agents can be neutralized by compounds present in the culture medium itself, such as the polyphenols that the AE could provide [56]. In the case of the yeast strains (Figure 6), no effect was observed on the viability of the strain *B. bruxellensis* LAMAP2480.

However, a decrease in viability was observed for *B. bruxellensis* LAMAP1359 (0.3 log cycle). For *B. bruxellensis* CECT1451 and *P. guilliermondii* NPCC1051, the AE had a favorable effect on cell viability compared to the control sample (Figure 6c,d). This phenomenon could be attributed to the presence of secondary metabolites originating in the plant world that were nutrients for these yeasts. One of the main groups synthesized in large quantities corresponds to phenolic compounds, which in some cases are essential for plant physiological functions, and others participate in defense functions against stress situations and various stimuli [6].

## 3. Conclusions

Our study indicates that the aqueous extract of *S. polygamus* (Cav.) Cabrera presents antimicrobial activity against *E. coli* ATCC25922, *L. monocytogenes* ATCC13932, and *S. Typhimurium* ATCC14028, reducing growth kinetics; however, it failed to completely inhibit cell functionality, observing a decrease of 1.5 logarithmic cycles on average. Regarding its antifungal action, it was determined that the AE affected the growth parameters of contaminating yeast strains *B. bruxellensis* and *P. guilliermondii*, prolonging their *lag* phase, thus increasing their generation time and decreasing their growth rate. This effect can be attributed to the presence of different phenolic compounds and flavonoids in the aqueous solution.

The main compounds found in the AE were *p*-coumaric acid, 4-vinylphenol, 4-ethylphenol, ferulic acid, and phenols such as naringenin and digallate have been described to have antimicrobial and antifungal properties. This antimicrobial activity is related to a decrease in microbial extracellular pH, as well as to the reduction of the lipid bilayer. However, research must continue to characterize the bioactive substances present, in addition to experiments to determine their efficiency and efficacy for future applications.

## 4. Material and Methods

### 4.1. Microorganism

All microorganisms tested in this study (Table 4) were obtained from the collection of the Laboratory of Biotechnology and Applied Microbiology (LAMAP) at the University of Santiago de Chile (Santiago, Chile).

Bacterial cultures were grown at 37 °C in 2.5% peptone water to a concentration of 107–108 CFU/mL. Yeasts were grown in YPD medium (0.5% peptone, 0.5% yeast extract, and 2% glucose) at 28 °C until saturation. The cell concentration was determined by the total cell count in the Neubauer chamber, according to the manufacturer’s specifications (Neubauer Improve Chamber, Precicolor, HBG, Umkirch, Germany).

### 4.2. Vegetal Material

The leaves of *S. polygamus* were collected from the trees between April and July 2016, in the town of El Corral, San Félix Valley, Alto del Carmen commune, Huasco province, located in the Atacama region, North of Chile (39°59′5.57″ S, 73°22′17.47″ O). This was selected based on popular use, and no chemotaxonomic criteria were used. The specimen was identified by the Herbarium of the Plant Sciences Department (UC), which was deposited under voucher number HDCV-2795-24.

### 4.3. Preparation of Extract

Leaves free of branches and fruits were washed with distilled water and disinfected by immersion in 2% sodium hypochlorite for 30 min. They were then rinsed thoroughly with distilled water. The leaves were dried at room temperature and then crushed. The extract was made according to popular usage and following the methodology proposed by Rhouma et al. [5]. Ten grams of dried leaf powder was mixed with 100 mL of distilled water at boiling temperature for 15 min with constant stirring to obtain a 10% *w*/*v* solution. Subsequently, filtration was performed through four layers of gauze and then through Whatman^®^ # 1 filter paper (Whatman International Ltd., Buckinghamshire, UK). The filtrate was stored at 4 °C until lyophilization concentration to 100X.

### 4.4. Quantification of Classic Antimicrobial Compounds in Natural Plants

#### 4.4.1. Hydroxycinnamic Acids

The concentration of the hydroxycinnamic acids and their derivatives was quantified using the method described by Ross et al. [57]. The HPLC technique was performed using Shimadzu Corporation (Tokyo, Japan), model Prominence equipment. The column used was the Shimadzu Inertsil ODS-3 (150 × 4.6 mm). The column was eluted at 40 °C with a solvent system corresponding to a water/acetic acid (90:10% *v*/*v*) and methanol gradient and a flow rate of 1 mL/min. A Shimadzu SPD-M20A detector at 320 nm (hydroxycinnamic acids) and 280 nm (volatile phenols) was used. All the standard compounds were obtained from Sigma-Aldrich Chemical Co. (St. Louis, MO, USA).

#### 4.4.2. Analysis of Phenolic Content

Phenolic compounds were analyzed according to Junqueira-Gonçalves et al. [58]. Liquid chromatography–mass spectrometry (LC/MS) analysis was performed using an Agilent 1100 Series Liquid Chromatograph/Mass Selective Detector equipped with a Quadrupole Mass Spectrometer (G6400) (Agilent Technologies, Palo Alto, CA, USA). The HPLC was compounded by a quaternary pump, online vacuum degasser, and thermostatic column compartment, connected in line to a mass spectrometer. The analysis was conducted in a column. A binary gradient of 1% formic acid and deionized water and acetonitrile was used with a flow rate of 1 mL/min. The mass spectrometer was fitted to an atmospheric pressure electrospray ionization source, operated in negative ion mode. The HPLC standards used for analysis and quantification were gallic acid, quercetin, rutin, chlorogenic acid, caffeic acid, syringic acid, myricetin, ellagic acid, kaempferol, naringenin, isoquercitrin, morin, genistein, and luteolin purchased from Sigma-Aldrich Chemical Co. (St. Louis, MO, USA).

### 4.5. Antimicrobial Activity against Bacteria and Yeasts

To determine the effect of the extract on the growth of microorganisms, growth curves were extracted from the microplate using a Sunrise reader (TECAN, Austria) coupled with the Magellan 7.2 program (TECAN, Grödig, Austria).

Bacteria were inoculated in 2.5% peptone water as the base culture medium at 1 × 10^4^ cells. A volume of 20 μL of aqueous extract of *S. polygamus* leaves (AE) corresponding to 10% of the final culture volume was added. The same volume of sterile distilled water and ampicillin 0.1 mg/mL were used in other cultures as an aqueous control (AC) and death control, respectively. The plate was incubated for two days at 37 °C.

In the case of the yeasts, these were inoculated in a YPD medium at a concentration of 1 × 10^5^ cells. A volume of 20 μL of aqueous extract of *S. polygamus* leaves (AE) corresponding to 10% of the final culture volume was added. The same volume of sterile distilled water and hygromycin 100 mg/mL were used in other cultures as an aqueous control (AC) and death control, respectively. The plate was incubated for three days at 28 °C.

### 4.6. Minimum Inhibitory Concentration (MIC)

Minimum inhibitory concentrations (MICs) were determined by the agar well diffusion method [59]. Concentrations of 5, 2.5, and 1.25% were prepared by two-fold serial dilution. One mL of each prepared inoculum was pipetted into sterile Petri dishes, followed by adding molten agar and mixed well. Then, six wells were made on each plate, and 100 μL of 5, 2.5, and 1.25% of each extract was transferred to the wells. Plates were incubated for 18 h at 37 °C for bacteria and 28 °C for yeast. The MIC was the lowest concentration inhibiting the respective microorganisms’ growth. All assays were performed in triplicate. Ethanol was used as a positive control, and distilled water was used as a control for extracts.

### 4.7. Evaluation of Cellular Viability of Microorganisms

Evaluation of cellular viability was performed by agar counting. Bacteria were inoculated in 2.5% peptone water medium at a concentration of 1 × 10^4^ cells. Then, 200 μL AE was added, corresponding to 10% of the total volume. AC and ampicillin were used as an aqueous control and death control, respectively. The tubes were incubated at 37 °C for 24 h. Viable cell count was performed on LB agar (0.5% yeast extract, 1% tryptone, 1% NaCl, and 2% agar) after 40 h of incubation.

In the case of yeasts, the same procedure was followed using YPD broth and hygromycin as an aqueous control and death control, respectively. Tubes were incubated at 28 °C for 48 h. Viable cells were counted on YPD agar (0.5% peptone, 0.5% yeast extract, 2% glucose, and 4% agar) after 72 h of incubation.

### 4.8. Statistical Analysis

Data were analyzed using the Statgraphics Centurion XVI.I program (Statpoint Technologies, Warrenton, VA, USA), using the multiple range test for data with a normal distribution. The existence of significant differences was considered when *p* ≤ 0.05.

## Figures and Tables

**Table 1:** Concentration of hydroxycinnamic acids and their derivatives present in the aqueous extract of *S. polygamus* 100X.

Compounds	Concentration (mg/L)
Ferulic Acid	223.95 ± 7.3
*p*-Coumaric acid	532.1 ± 0.3
Caffeic Acid	ND
4-Vinylphenol	370.1 ± 8.6
4-Vinylguaiacol	ND
4-Ethylphenol	281.55 ± 36.1
4-Ethylguaiacol	ND

ND: not detected.

**Table 2:** Concentration of phenolic compounds in higher concentration present in aqueous extract of *S. polygamus* 100X.

Compounds	Concentration (mg/mL)
**Naringenin 4′-O-glucoside**	1.19 ± 0.007
**Genistin 6″-O-acetate**	0.57 ± 0.074
**Myricetin 7-glucoside**	0.74 ± 0.084
**2′,6′-Dihydroxy-4′-methoxy-3′-(2-hydroxybenzyl)dihydrochalcone**	1.49 ± 0.21
**Digallate**	1.18 ± 0.12
**Quercetin 3′-glucoside**	0.15 ± 0.050
**1′-O-Galloylsucrose**	0.79 ± 0.13
**1,2-Digalloyl-beta-D-glucopyranose**	0.68 ± 0.053
**Salicylic acid**	0.058 ± 0.006
**5,7-Dihydroxy-3′,4′-dimethoxy-5′-prenylflavanone**	0.43 ± 0.025
**(E)-2-O-Cinnamoyl-beta-D-glucopyranose**	0.051 ± 0.013
**3,5,7,3′,5′-Pentahydroxy-6,4′-dimethoxyflavone**	0.044 ± 0.002
**Jamaicin**	0.044 ± 0.002
**3,5,6,7-Tetrahydroxy-4′-methoxyflavone**	0.081 ± 0.007
**Luteolin 5,7,3′,4′-tetramethyl ether**	0.27 ± 0.005
Rutin	0.45 ± 0.0023
**Rhamnetin 3-glucoside**	0.19 ± 0.002
Ellagic acid	0.16 ± 0.013
**2,6-Digalloylglucose**	0.39 ± 0.100
**Phloridzin**	0.14 ± 0.016

Putative compounds in bold.

**Table 3:** Minimum inhibitory concentration of aqueous extract of *S. polygamus* 100X.

Microorganism	Minimum Inhibitory Concentration (%).
*E. coli* ATCC25922	5
*S. Typhimurium* ATCC14028	>5
*L. monocytogenes* ATCC13932	5
*B. bruxellensis* LAMAP2480	>5
*B. bruxellensis* LAMAP1359	2.5
*B. bruxellensis* CECT1451	>5
*P. guilliermondii* NPCC1051	>5

**Table 4:** Microorganisms used in this study.

Microorganisms	Specie	Code
Yeast	*Brettanomyces bruxellensis*	LAMAP2480 *
Yeast	*Brettanomyces bruxellensis*	LAMAP1359 *
Yeast	*Brettanomyces bruxellensis*	CECT1451
Yeast	*Pichia guilliermondii*	NPCC1051
Bacteria	*Escherichia coli*	ATCC25922
Bacteria	*Salmonella Typhimurium*	ATCC14028
Bacteria	*Listeria monocytogenes*	ATCC13932

* *Indigenous strains*; ATCC—American Type Culture Collection; CECT—Colección Española de Cultivos Tipo; NPCC—North Patagonian culture collection.

## Data Availability

The datasets presented in this article are not readily available because are part of an ongoing study. Requests to access the datasets should be directed to corresponding author on reasonable request.

## Supplementary Materials

The following supporting information can be downloaded at: https://www.mdpi.com/article/10.3390/plants13162248/s1, Table S1: Determination of the lag phase, specific growth rate, generation time, and percentage of inhibition.
