# Supplementary material for: Antimicrobial Activity of Leaf Aqueous Extract of Schinus polygamus (Cav.) Cabrera against Pathogenic Bacteria and Spoilage Yeasts
Source: Plants (Basel). 2024 Aug 13;13(16):2248. doi: 10.3390/plants13162248 (PMC11360385; doi:10.3390/plants13162248)
Supplement: Supplementary file 1 [file plants-13-02248-s001.zip › plants-3125166-supplementary.pdf]

Table S1. Determination of the lag phase, specific growth rate, generation time, and percentage of inhibition.

	***Lag* phase (h)**		**μ max (h^{-1})**		**Tg (h)**		**Inhibition percentage (%)**
**Strain**	**Control**	**AE**	**Control**	**AE**	**Control**	**AE**	
E. coli	5.00±0.00	-	1.024±0.009	-	0.68±0.008	-	-
L. monocytogenes	7.57±0.706	-	0.035 ± 0.008	-	29.14 ± 7.22	-	-
S. Typhimurium	6.10±1.91	-	0.045 ± 0.002	-	22.32 ± 1.09	-	-
***B. bruxellensis* LAMAP2480**	27.00 ±1.86[a]	41.96±12.34[b]	0.051 ± 0.001[a]	0.006 ± 0.002[b]	19.61 ± 0.61[a]	174.85 ± 49.99[b]	88.23
***B. bruxellensis* LAMAP1359**	58.51±5.62[a]	69.5±10.87[a]	0.025 ± 0.002[a]	0.0045 ± 0.0004[b]	38.77 ± 0.002[a]	223.40 ± 19.93[b]	82.62
***B. bruxellensis* CECT1451**	43.98±1.58[a]	58.68±10.74[b]	0.048 ± 0.0003[a]	0.007 ± 0.001[b]	20.77 ± 0.15[a]	146.31 ± 26.14[b]	85.42
***P. guilliermondii* NPCC1051**	8.89±0.29[a]	51.17±8.58[b]	0.042 ± 0.002[a]	0.013 ± 0.003[b]	23.69 ± 1.17[a]	82.95 ± 30.082[b]	69.04

Different letters in superscript indicate significant differences (P<0.05) for each kinetic parameter analyzed.
